# Androgen Receptor Signaling in Bladder Cancer

**DOI:** 10.3390/cancers9020020

**Published:** 2017-02-22

**Authors:** Peng Li, Jinbo Chen, Hiroshi Miyamoto

**Affiliations:** 1 Department of Pathology, Johns Hopkins University School of Medicine, Baltimore, MD 21287, USA; 001lipeng@163.com; 2 Department of Urology, Johns Hopkins University School of Medicine, Baltimore, MD 21287, USA; 3 Minimally Invasive Urology Center, Shandong Provincial Hospital Affiliated to Shandong University, Jinan 250021, China; 4 Department of Pathology & Laboratory Medicine, University of Rochester Medical Center, Rochester, NY 14642, USA; jinbo_chen@urmc.rochester.edu; 5 James P. Wilmot Cancer Center, University of Rochester Medical Center, Rochester, NY 14642, USA; 6 Department of Urology, Xiangya Hospital of Central South University, Changsha 410008, China; 7 Department of Urology, University of Rochester Medical Center, Rochester, NY 14642, USA

**Keywords:** androgen, androgen receptor, anti-androgen, carcinogenesis, tumor progression, urothelial cancer

## Abstract

Emerging preclinical findings have indicated that steroid hormone receptor signaling plays an important role in bladder cancer outgrowth. In particular, androgen-mediated androgen receptor signals have been shown to correlate with the promotion of tumor development and progression, which may clearly explain some sex-specific differences in bladder cancer. This review summarizes and discusses the available data, suggesting the involvement of androgens and/or the androgen receptor pathways in urothelial carcinogenesis as well as tumor growth. While the precise mechanisms of the functions of the androgen receptor in urothelial cells remain far from being fully understood, current evidence may offer chemopreventive or therapeutic options, using androgen deprivation therapy, in patients with bladder cancer.

## 1. Introduction

Urinary bladder cancer, mostly urothelial carcinoma, is the second most common genitourinary malignancy, with an estimate of 429,800 new cases and 165,100 deaths in 2012 worldwide [1]. Despite significant advances in diagnostic technologies as well as surgical techniques and adjuvant/neoadjuvant treatment strategies, the prognosis of patients with bladder cancer has remained largely unchanged over the last few decades. Thus, patients with a non-muscle-invasive bladder tumor still carry a life-long risk of recurrence with occasional progression to muscle invasion following transurethral surgery, while those with a muscle-invasive tumor are at a high risk of metastasis following radical cystectomy. Indeed, current non-surgical conventional treatments, such as intravesical pharmacotherapy and systemic chemotherapy, do not result in complete prevention of tumor recurrence or significant reduction in mortality [2,3]. Of note, mainly due to the life-long need for monitoring for recurrence, bladder cancer has been reported to have the highest lifetime costs per patient among all malignancies [4,5]. As a result, further studies are urgently needed to better understand the molecular mechanisms for bladder cancer development and progression, which may not only provide effective targeted therapy but also contribute to the reduction of treatment costs.

Men are at a significantly higher risk of bladder cancer than women in the US as well as virtually all countries/regions, while there is an approximately 10-fold variation in its incidence internationally [1,6]. Cigarette smoke and exposure to industrial work-related chemicals—well-established risk factors for bladder cancer—were thought to contribute to the sex-disparity. However, men are still 3–4 times more likely to develop bladder cancer than women even after controlling these environmental or lifestyle factors [1,6,7,8]. Accordingly, intrinsic factors are likely to play a critical role in urothelial carcinogenesis. Meanwhile, preclinical evidence has strongly suggested the involvement of androgen receptor (AR) signaling in bladder tumorigenesis and cancer progression.

Previous studies have thus demonstrated that AR activation generally correlates with the promotion of the development and growth of urothelial cancer. In this article, we review these available data and highlight underlying molecular mechanisms.

## 2. Androgens, AR Signaling, and Their Physiological Functions in the Bladder

Androgens, first discovered in 1936, are a class of steroid hormones, mainly secreted by the testis, ovary, and adrenal cortex. These include testosterone and its metabolite via 5α-reductase in certain tissues, dihydrotestosterone (DHT), as well as adrenal androgens, dehydroepiandrosterone, androstenediol, and androstenedione. In males, androgens can stimulate the differentiation and maturation of the sex organs and the development of secondary sex characteristics as well as maintain sexual activity and reproductive function [9,10]. The physiological functions of androgens are mainly dependent on their binding to AR in target cells to stimulate a series of post-receptor biochemical changes [9,10,11,12].

The AR, a 110 kD protein composed of 919 amino acids, is a member of the nuclear receptor superfamily that functions as a ligand-inducible transcription factor and mediates the biological effects of androgens in a wide range of physiological and pathological processes [11,12,13]. The human *AR* gene locates on the X chromosome (Xq11–12) and contains eight exons and seven introns with the total length exceeding 90 kb. The AR encodes four distinct functional domains: the N-terminal transactivation domain, the DNA-binding domain (DBD), a hinge region, and the C-terminal ligand-binding domain (LBD) [13]. It usually locates in the cytoplasm coupling with heat shock proteins. Upon binding of androgens at the LBD, AR is released from heat shock proteins and is translocated into the nucleus in the form of a phosphorylated homodimer. Then, AR binds to androgen response elements (AREs) in the genome as well as to a variety of co-regulators, leading to a series of specific activation or repression of gene transcription [13,14]. An alternative mechanism of AR activation independent of androgen binding includes its phosphorylation via kinases [e.g., epidermal growth factor receptor (EGFR)] in, for instance, prostate cancer cells [15,16,17]. Truncated AR isoforms that lack the LBD have also been found and are constitutively active in the absence of androgens [18].

Male internal genitalia, including the prostate and bulbourethral gland as well as urothelium, are derived from the urogenital sinus endoderm. Simultaneously, it is well known that the differentiation of the prostate and its development require the induction of AR signaling [19]. Thus, we can infer that AR signaling also contributes to bladder development. Meanwhile, AR expression has been documented in a variety of human or rodent tissues [20,21]. AR has also been found to be present in urothelium as well as bladder submucosa, such as smooth muscle cells and neurons [20,21,22,23,24]. However, physiological functions of AR in some of the organs, including the bladder, remain far from being fully understood. Animal studies have shown that AR is involved in the regulation of urine storage and urinary tract functions. Castration in male animals resulted in significant decreases in the activity and expression of tissue enzymes closely related to cholinergic and non-cholinergic nerve functions [25,26]. Androgen supplementation in castrated male rats also re-augmented the thickness of urothelium, the quantity of smooth muscle fibers, and the number of vessels in their bladders [27]. In addition, androgen deficiency was found to induce bladder fibrosis and reduce the bladder capacity and compliance in male rats [28]. Thus, androgens appear to contribute to improving/maintaining bladder functions. It has indeed been shown in a few clinical studies that testosterone treatment is beneficial to men with lower urinary tract symptoms [29,30]. Conversely, testosterone was shown to inhibit neurogenic and chemogenic responses in the rat bladder, resulting in the reduction of detrusor muscle contraction [31]. To the best of our knowledge, there are no recent clinical studies further assessing the efficacy of androgen treatment in those with lower urinary tract symptoms.

## 3. Alterations of AR in Bladder Cancer

Prior to its cloning, a binding assay suggested higher levels of AR content in bladder tumor (49.5 Fm/mg) than in normal bladder mucosa (17.2 Fm/mg), as well as in male (68.0 Fm/mg) or low-grade (43.8 (male)/27.7 (female) Fm/mg) tumors than in female (27.7 Fm/mg) or high-grade (32.4 Fm/mg) tumors, respectively [32]. Thereafter, immunohistochemical studies in surgical specimens have assessed the expression status of AR in different grades/stages of bladder tumors, in comparison with normal/non-neoplastic urothelial tissues in some of them [33,34,35,36,37,38,39,40,41,42,43,44] (Table 1). Of note, a PCR-based method could detect the *AR* gene in all 33 superficial bladder cancer specimens examined [45].

The positive rates of AR expression immunohistochemically detected in bladder tumor tissues involving more than 40 cases range from 13% to 55%, which are significantly lower than those in non-neoplastic urothelial samples in some studies [34,36,40]. In contrast, at least two studies have demonstrated no detectable AR in normal urothelial tissues examined [38,42]. These conflicting findings may have resulted from differences in tissue preservation (e.g., formalin fixation), staining protocol (e.g., antibody), and/or signal scoring. In addition, the so-called cancer field effect may have affected the immunoreactivity because normal-appearing tissues from patients with bladder cancer were used in most of these studies. Nonetheless, these immunohistochemical studies have failed to reveal significant sex-related differences in AR expression in male versus female tissues (normal, tumor). A significant decrease in the AR positive rate was also reported in urothelial carcinomas of the upper urinary tract, compared with corresponding non-neoplastic urothelial tissues [46].

Some of these studies have compared the rates of AR positivity in low-grade or non-muscle-invasive tumors versus high-grade or muscle-invasive tumors. Similar to the AR positivity in bladder tumors compared with non-neoplastic bladders, its significant or insignificant down-regulation is observed in high-grade and/or muscle-invasive tumors [34,35,36,38,40,43]. Similar findings were observed in upper urinary tract tumors [46,47,48]. Thus, AR expression appears to be down-regulated or lost during steps of tumorigenesis and tumor progression in spite of the promoting effects of AR signals as described below. In contrast, a few other studies showed slight increases in AR positivity in high-grade and/or muscle-invasive bladder tumors [37,41].

Prognostic values of AR expression in bladder cancer patients have also been assessed, and the findings remain controversial. Two studies indicated a correlation between AR positivity and a lower risk of tumor recurrence [38,43]. Meanwhile, AR expression was shown to correlate with the risk of tumor progression [40,42]. Other studies have failed to show prognostic significance of AR expression in bladder or upper urinary tract tumors [36,37,46,47]. It has also been suggested that muscle-invasive bladder cancers are initially androgen-sensitive for their growth, which is eventually lost due to the activation of certain genes possessing an ARE in their promoter region in an androgen-independent manner—as seen in prostate cancer—and induces metastatic potential of tumor cells [49]. Thus, AR expression may not necessarily serve as a prognosticator in patients with bladder cancer.

In addition to the differential expression of AR protein, genetic alterations involving the *AR* gene have been documented in bladder cancer. Loss of heterozygosity at the AR locus was identified in muscle-invasive tumors and concurrent lesions of carcinoma in situ from female patients [50]. In addition, several studies have demonstrated differences in the number of polyglutamine (CAG) repeats within exon 1 of the *AR* gene, which in general is inversely correlated with its transcriptional activity, between bladder tumors and controls or different grades/stages of bladder tumors. Men and women who had 23 (odds ratio = 2.09) and 44 (cumulative; odds ratio = 4.95) CAG repeats were found to have a significantly elevated risk of urothelial carcinoma, compared to those with longer CAG [51]. A significantly shorter CAG repeat length was also identified in 95 male patients with bladder cancer (mean: 19.8), compared with 94 control males (mean: 21.1) [52]. Moreover, there appeared to be a link between shorter CAG repeat length and more aggressive features of bladder cancer in a relatively small number of cases [53]. Short CAG repeat lengths (20 in UMUC3 and 22 in TCCSUP) were also identified in two AR-positive human bladder cancer cell lines [35]. Meanwhile, although no somatic mutations in the *AR* gene were found in 99 cases of bladder cancer [54], a molecular profiling data search [55,56] identified them in up to 4% (2 of 50) of urothelial carcinomas of the bladder as well as in 6.1% (2 of 33) of plasmacytoid urothelial carcinomas. AR isoforms (i.e., 90 kDa, 60 kDa) were also detected in some of tumor specimens [33], suggesting the presence of its splice variants in bladder cancer.

## 4. Role of AR Signaling in Urothelial Carcinogenesis

The gender-specific difference in the incidence of bladder cancer as well as AR expression in benign and cancerous urothelium suggests the involvement of AR signaling in urothelial tumorigenesis. High incidence of high-grade prostatic intraepithelial neoplasia and prostatic adenocarcinoma—in the development of both of which, AR plays a critical role—in cystoprostatectomy specimens undergone for bladder urothelial carcinoma (e.g., 24.4% for the latter in a meta-analysis involving 13,140 patients [57]) may also support the presence of common tumorigenesis signals between these two malignancies. Based on these observations, previous studies using various approaches have assessed the role of androgens and/or AR in urothelial carcinogenesis.

A chemical carcinogen, *N*-butyl-*N*-4-hydroxybutyl nitrosamine (BBN), which is known to induce a bladder tumor effectively in experimental rodents and more rapidly in male animals than in females [58], has been used to assess the effects of androgens, AR, and anti-AR treatment on bladder carcinogenesis. In 1975, it was shown that testosterone treatment in female rats increased the incidence of BBN-induced bladder tumors while a synthetic estrogen diethylstilbestrol in males decreased it [59]. In 1997, hormonal treatment with a gonadotropin-releasing hormone analogue as chemical castration or an anti-androgen flutamide was shown to prevent the development of BBN-mediated tumors in male rats [60]. Subsequently, BBN was found to fail to induce bladder cancer in male or female AR knockout (ARKO) mice [45]. Testosterone treatment and surgical orchiectomy were also shown to increase and decrease, respectively, the incidence of bladder tumors in male rats with administration of another carcinogen *N*-nitrosobis(2-oxopropyl)amine [61]. Thus, androgen-mediated AR signals appeared to play a critical role in bladder carcinogenesis induced by chemical carcinogens. However, a subset of male ARKO mice treated with BBN and supplemented with DHT developed bladder tumors [45], suggesting the involvement of androgen-mediated non-AR pathways in bladder tumorigenesis. Otherwise, because only DBD in exon 2 of the *AR* gene was disrupted in the ARKO mice [62], the androgen effect on bladder tumorigenesis might be mediated through the truncated AR protein that is unable to bind to DNA. An additional possibility was that the second zinc finger of the DBD in exon 3 had residual DNA binding activity. More recently, BBN was also found to fail to induce bladder tumors in male mice having normal levels of testosterone yet lacking AR specifically in the urothelium [63]. Similarly, the incidence of a BBN-induced bladder tumor in a transgenic mouse model where AR is conditionally expressed in the bladder urothelium was higher than that in age and sex matched controls [64]. In addition, castration inhibited the development of bladder tumors in another transgenic mouse model in which constitutive active β-catenin in the urothelial basal cells spontaneously induced high-grade urothelial cancer [65]. These observations further suggest a critical role of urothelial AR, but not ARs in other organs, in bladder carcinogenesis.

Several recent retrospective cohort studies have supported these findings in animals indicating that AR activation correlates with the induction of bladder tumorigenesis. First, men undergoing androgen deprivation therapy (ADT) for their prostate cancer were shown to have a considerably lower risk of subsequent development of bladder cancer (0/266 (0%)), compared with those undergoing surgery alone (5/437 (1.1%)) or radiotherapy (14/631 (2.2%)) [66]; second, in 162 men with a history of prostate and bladder cancers, ADT used for the treatment of the former strongly prevented the recurrence of the latter, compared with those without ADT [67]. In this cohort, AR expression in their bladder tumors was also found to be an independent predictor of the preventive effects of ADT on tumor recurrence [68]; third, in 228 men with a history of bladder cancer, ADT (for their prostate cancer) or a 5α-reductase inhibitor dutasteride treatment (for their benign prostatic hyperplasia) resulted in significant reduction in the rate of bladder tumor recurrence, compared with 196 control patients without hormonal treatment [69]; finally, in a prospective cohort study involving 72,370 men, treatment with a 5α-reductase inhibitor finasteride primarily prescribed for their symptomatic benign prostatic hyperplasia significantly reduced the risk of bladder cancer development (hazard ratio = 0.634; *p* = 0.0004) [70], although a preclinical study failed to show a significant inhibitory effect of finasteride on a BBN-induced bladder tumor [60].

Androgens have been shown to modulate the activity and/or expression of certain enzymes via the AR pathway. These enzymes include cytochrome P450 (e.g., CYP4B1) and UDP-glucuronosyltransferase (e.g., UGT1A subtypes) that are known to involve the activation and detoxification, respectively, of bladder carcinogens, such as aromatic amines. The levels of *CYP4B1* gene expression in male mouse bladders were found to be higher than those in female mouse bladders, and castration in males resulted in a decrease in its expression [71]. Similarly, the expression levels of mouse Ugt1a subtypes were elevated in the bladders from intact female or ARKO male mice, compared with those from intact/control male mice [72]. In addition, orchiectomy [72] or ovariectomy [73] up- or down-regulated, respectively, the expression of some Ugt1a subtypes in the mouse bladders. Meanwhile, in SVHUC human normal urothelial cells stably expressing wild-type full-length AR, DHT treatment resulted in considerable decreases in the expression of all UGT1A subtypes, and an anti-androgen hydroxyflutamide blocked the DHT effects [72]. Moreover, in a mouse model, castration was shown to reduce bladder susceptibility to a carcinogen 4-aminobiphenyl via modulating UGT1A3 in the liver [74].

GATA3 is a zinc-finger transcription factor and is highly expressed in urothelial cells. Loss of GATA3 expression in a subset of bladder cancers, especially high-grade and/or muscle-invasive tumors [75], as well as its correlation with the induction of tumor cell migration and invasion in vitro [76], suggests the role of GATA3 as a tumor suppressor. Indeed, in an in vitro transformation model using SVHUC cells, GATA3 silencing resulted in the induction of malignant transformation as well as down- or up-regulation of the expression of tumor suppressors (e.g., p53, p21, p27, PTEN, UGT1A) or oncogenic molecules (e.g., c-myc, cyclin D1/D3/E, FGFR3), respectively [77]. In SVHUC sublines with or without undergoing neoplastic transformation induced by carcinogen challenge, AR overexpression or androgen treatment considerably reduced GATA3 expression [77]. In addition, orchiectomy increased and ovariectomy decreased the levels of GATA3 expression in the mouse bladders [77]. Thus, in non-neoplastic urothelial cells, AR activation appears to correlate with the down-regulation of the expression of GATA3 that prevents neoplastic transformation.

## 5. Role of AR Signaling in Urothelial Cancer Progression

In addition to its involvement in urothelial carcinogenesis, there have been a variety of studies suggesting that androgens and/or AR promote bladder cancer progression. As seen in prostate cancer cells, androgens could induce AR expression and its nuclear translocation as well as ARE promoter activity in bladder cancer cells [39,41,45,78,79,80,81,82,83]. In some of these studies, AR antagonists, such as flutamide, bicalutamide, and enzalutamide, were shown to block the effects of androgens on AR expression or transcription.

Using cell viability or colony formation assays, androgens have been shown to induce the growth of AR-positive bladder cancer cells [39,45,79,80,82,83,84,85,86,87,88,89]. Accordingly, AR knockdown as well as treatment with AR antagonists inhibited the cell proliferation of bladder cancer lines cultured with androgens. In an earlier study using the R198 transplantable bladder cancer line, tumor growth in male mice was facilitated by DHT administration [90]. Subsequent studies using mouse xenograft models for bladder cancer demonstrated that orchiectomy or treatment with anti-AR compounds could considerably inhibit tumor growth [41,45,84,86,87,88,91]. In a transgenic mouse model expressing SV40 large T antigen specifically in urothelium (via uroplakin II) and spontaneously developing bladder cancer, castration after tumor formation retarded its growth, which was restored by DHT supplement [92]. Similarly, in vitro assays have demonstrated that androgen-mediated AR signals promote the migration and invasion of bladder cancer cells [41,82,83,88]. Then, AR knockdown or anti-androgen treatment was shown to inhibit them [41,83,86,87,88]. Additionally, in the uroplakin II-SV40T transgenic model, castration reduced microvessel density in bladder tumors and increased the expression of an anti-angiogenic factor TSP-1 [92], indicating the promotion of angiogenesis by AR activation in bladder cancer.

In AR-positive bladder cancer cells, androgens are able to modulate the expression or activity of various molecules/pathways. Those known to involve bladder cancer cell proliferation/migration/invasion as well as angiogenesis/metastasis include β-catenin/Wnt signaling and its downstream targets c-myc/cyclin D1 [65,81,84,86], CD24 [80,88], EGFR family and its downstream AKT/ERK [39,79], ELK1 [82], matrix metalloproteinases (MMPs) [45,65,83,86,88,93], and vascular endothelial growth factor [45,88]. Androgen-mediated AR signals were also shown to induce epithelial-to-mesenchymal transition via modulating the expression of Slug and the activity of β-catenin/Wnt signaling in bladder cancer cells [41,87]. More recently, in vitro assays demonstrated that bladder cancer cells could recruit B cells [94], T cells [95], and neutrophils [93], leading to the induction of cell invasion as well as the expression of AR and MMPs. These observations may represent underlying molecular mechanisms for the promoting effects of androgens on bladder cancer progression.

As seen in prostate cancer cells, non-androgens, such as epidermal growth factor (EGF), could increase AR transcriptional activity in bladder cancer cells, which was blocked by AR antagonists [79]. EGF could also induce AR-positive bladder cancer cell proliferation in the absence of androgens [79,85]. More interestingly, EGF and DHT appeared to show synergistic effects on the proliferation as well as phosphorylation of EGFR, AKT, and ERK in bladder cancer cells [39,79].

Recent in vitro studies have suggested a correlation between AR activity in bladder cancer cells and chemosensitivity. AR-positive cell lines were more resistant to cisplatin than control AR-negative or AR knockdown cells cultured in the presence of androgens [96]. Furthermore, androgen or anti-androgen treatment resulted in a decrease or an increase, respectively, in sensitivity to cisplatin in AR-positive bladder cancer cells, presumably via modulating the activity of a key factor of cisplatin resistance NF-κB [96]. Similarly, bladder cancer cells overexpressing AR or those treated with DHT were found to be more resistant to doxorubicin, an anti-cancer agent often used for intravesical pharmacotherapy, than respective control cells [84]. However, there were no significant differences in sensitivity to 5-fluorouracil [84] or gemcitabine [96] between AR-positive versus AR-negative bladder cancer cells or between AR-positive cells with versus without androgen treatment. In addition, enzalutamide treatment or AR knockdown was shown to inhibit the growth of gemcitabine-resistant bladder cancer cells, while whether it could increase chemosensitivity was not tested [89]. Of note, in these studies, AR expression was shown to be considerably elevated in “resistant” cell lines after long-term culture with cisplatin [96], doxorubicin [84], or gemcitabine [89], compared with control lines.

## 6. AR Co-Regulators in Bladder Cancer

As aforementioned, androgen-mediated AR transcriptional activity can be further enhanced by co-activators. Indeed, several AR co-regulators have been implicated in the modulation of bladder cancer cell growth. A cross-talk between AR-co-regulators and other signaling pathways in bladder cancer cells may further promote urothelial tumorigenesis and tumor progression.

Immunohistochemistry in tissue samples showed the expression of NCOA1, NCOA2, NCOA3, CREBBP, and EP300, in 85%–100% of bladder tumors—some of which even lacked AR expression [35]. Furthermore, of these AR co-activators, only NCOA1 expression was significantly down-regulated in tumors, compared with non-neoplastic urothelial tissues. Meanwhile, knockdown of each co-activator led to significant reduction in cell proliferation of AR-positive bladder cancer lines, although, inconsistent with the findings in prostate cancer cells, androgen treatment failed to up-regulate the expression levels of these co-activators in these cells [35]. Therefore, distinct mechanisms may underlie co-regulator functions in bladder cancer versus other AR-positive malignancies such as prostate cancer.

Immunohistochemistry in radical cystectomy specimens also showed strong correlations of the expression of JMJD2A and LSD1, both of which were shown to mediate AR transactivation via histone-lysine demethylation mechanisms, with that of AR [36]. Moreover, significant down- and up-regulation of JMJD2A and LSD1, respectively, were found in bladder cancer specimens, compared with benign urothelial tissues. Loss of JMJD2A was also associated with lymphovascular invasion or worse overall survival, but not cancer-specific mortality. Remarkably, pharmacological inhibition of LSD1 resulted in significant decreases in the growth and androgen-induced AR transcription in bladder cancer cells [36].

Altered expression of β-catenin is well known to correlate with the progression of bladder cancer and poor patient outcomes [41,81,97]. Additionally, as described above, constitutive active β-catenin in mouse bladder cells could induce urothelial tumorigenesis [65]. In AR-positive bladder cancer cells, androgens have also been shown to activate β-catenin/Wnt signaling [65,81]. Moreover, AR and β-catenin co-express at the nuclei of bladder cancer cells and form a complex with T-cell factor, a co-factor of β-catenin and a downstream component of Wnt signaling, in the presence of androgens [81]. Thus, androgen-mediated AR signals appear to synergize with β-catenin in bladder cancer cells and may thereby promote tumor growth.

## 7. Concluding Remarks

**Figure 1:**
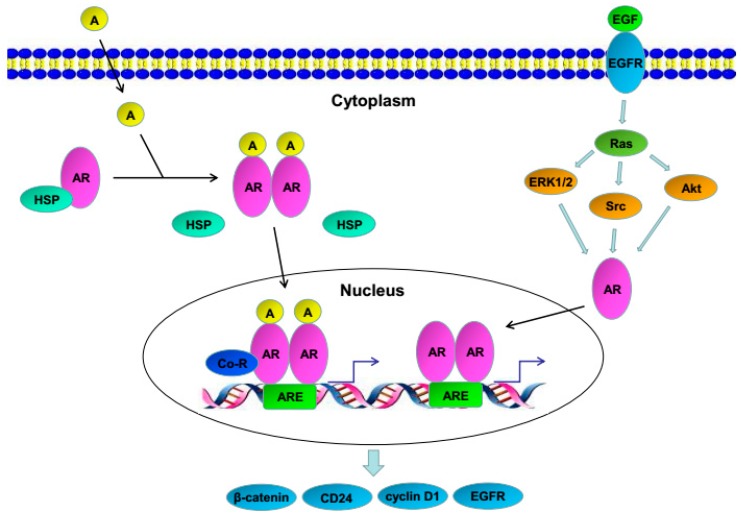
AR signaling in bladder cancer. A, androgen; AR, androgen receptor; ARE, androgen response element; Co-R, co-regulator; EGF, epidermal growth factor; EGFR, epidermal growth factor receptor; HSP, heat shock protein.

Current evidence indicating correlations of AR activation with the promotion of urothelial tumorigenesis and tumor progression supports that bladder cancer is a member of endocrine-related tumors. It is thus likely that at least AR and its associated signaling pathways, as depicted in Figure 1, play an important role in the pathogenesis of bladder cancer, which also helps explain the sex disparities, especially its incidence between men and women. However, underlying mechanisms of how AR and related signals regulate bladder cancer outgrowth still need to be elucidated. It also remains unclear whether androgen-mediated AR signals are the central pathway in modulating bladder carcinogenesis. Accordingly, further mechanistic studies are required to determine the precise functional role of AR signaling in the development and progression of bladder cancer.

Again, current non-surgical conventional treatments, such as intravesical pharmacotherapy and systemic chemotherapy, often fail to completely prevent the recurrence of superficial bladder tumors or significantly reduce the mortality rate in patients with advanced bladder cancer. Moreover, no approved targeted therapy for bladder cancer is available. As aforementioned, AR signals likely promote the development and progression of urothelial cancer. We therefore anticipate that AR inactivation—even via available options clinically used for the treatment of, for instance, prostate cancer—offers an effective chemopreventive or therapeutic approach for urothelial cancer. Indeed, two phase II clinical trials are being conducted to assess the preventive effects of enzalutamide on tumor recurrence in patients with non-muscle-invasive bladder cancer (NCT02605863) and the therapeutic effects of abiraterone—an androgen biosynthesis inhibitor prescribed in men with castration-resistant prostate cancer—in patients with advanced bladder cancer (NCT02788201). In addition, a phase I trial (NCT02300610) assessing the combination effects of enzalutamide with gemcitabine and cisplatin in patients with urothelial cancer is recruiting participants. Further prospective cohort studies of anti-AR treatment in patients with bladder cancer are thus encouraged.

## Figures and Tables

**Table 1:** Immunohistochemical studies showing correlations between androgen receptor (AR) expression in bladder cancer and clinicopathological features.

Study [Reference]	N	AR Positivity							
		Non-tumor vs. Tumor		Patient Gender		Tumor Grade		Tumor Stage	
		Non-tumor	Tumor	Male	Female	Low	High	NMI	MI
Zhuang et al., 1997 [33]	9	NA	44.4%	50.0%	33.3%	NA	NA	20.0%	75.0%
Boorjian et al., 2004 [34]	49	86.5%	53.1%	61.1%	30.1%	88.9%	48.5%	75.0%	21.4%
Boorjian et al., 2009 [35]	55	NA	43.6%	NA	NA	NA	NA	59.1%	33.3%
Mir et al., 2011 [37]	472	NA	12.9%	14.0%	8.1%	12.2%	13.1%	9.0%	15.1%
Tuygun et al., 2011 [38]	139	0%	51.1%	66.7%	61.5%	63.9%	37.3%	60.4%	21.2%
Zheng et al., 2011 [39]	24	NA	33.3%	NA	NA	40.0%	31.6%	NA	NA
Miyamoto et al., 2012 [40]	188	80.1%	42.0%	41.9%	42.5%	55.4%	36.4%	50.5%	33.0%
Jing et al., 2014 [41]	58	NA	53.4%	56.8%	42.9%	55.0%	50.0%	48.9%	69.2%
Mashhadi et al., 2014 [42]	120	0%	21.7%	NA	NA	NA	NA	NA	NA
Nam et al., 2014 [43]	169	NA	37.3%	38.5%	30.8%	39.2%	32.7%	NA	NA
Williams et al., 2015 [44]	297	NA	24.6%	NA	NA	NA	NA	33.6%	19.5%

N: number of cases; NMI: non-muscle-invasive; MI: muscle-invasive; NA: not applicable.
